# Supplementary material for: Positive Correlation of Peripheral CD8+ T Lymphocytes with Immune-Related Adverse Events and Combinational Prognostic Value in Advanced Non-Small Cell Lung Cancer Patients Receiving Immune Checkpoint Inhibitors
Source: Cancers (Basel). 2022 Jul 22;14(15):3568. doi: 10.3390/cancers14153568 (PMC9331848; doi:10.3390/cancers14153568)
Supplement: Supplementary file 1 [file cancers-14-03568-s001.zip › cancers-1800258-supplementary.pdf]

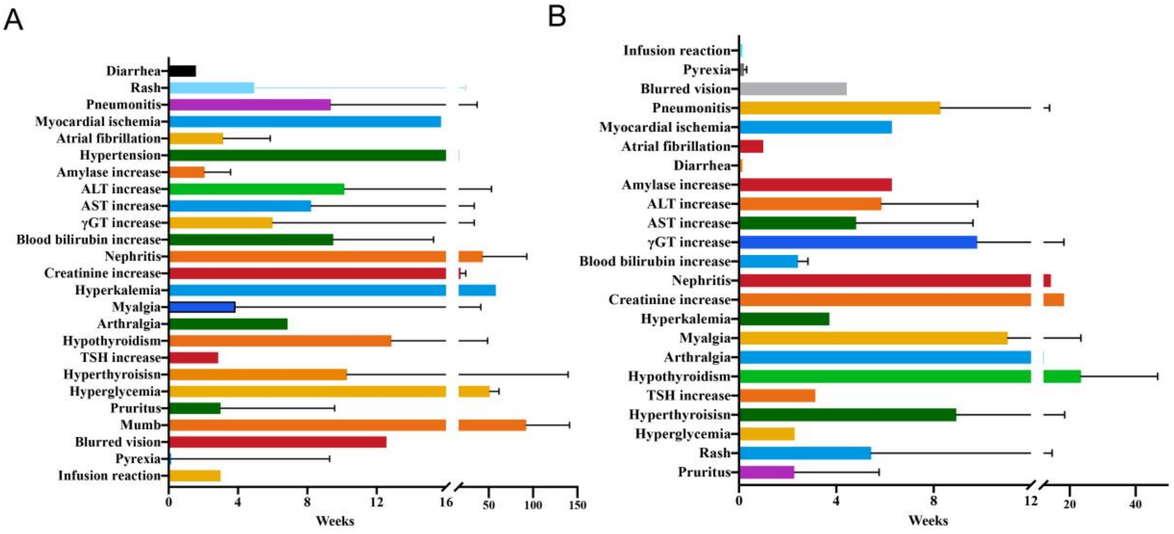

Supplementary figure S1. Onset and resolution time of immune-related adverse events (irAEs). (**A**) Onset time of different irAEs. (**B**) Resolution time of different irAEs. Abbreviation: ALT, alanine transaminase; AST, aspartate aminotransferase; γ GT, gamma-glutamyltransferase.

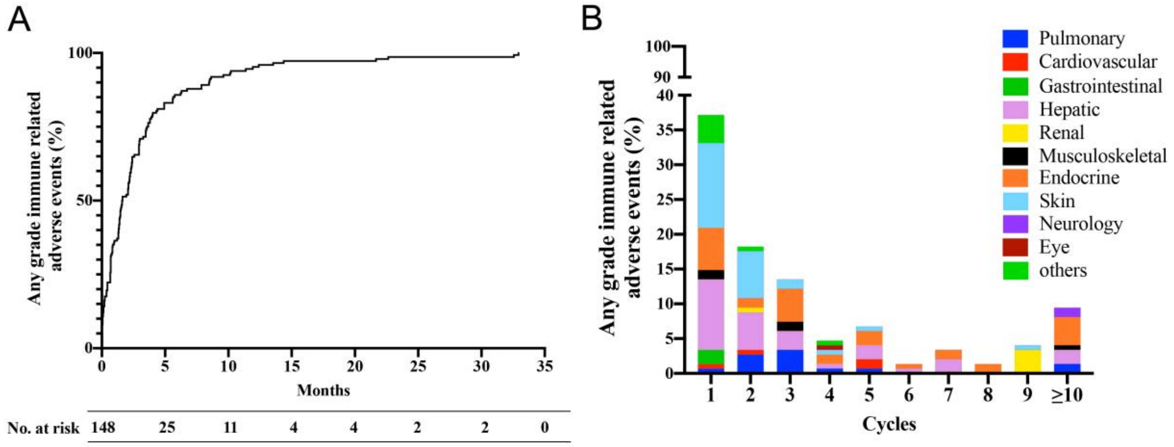

Supplementary figure S2. Any-grade immune-related adverse events (irAEs). (**A**) Time to first any-grade irAEs of 109 patients. (**B**) New any-grade irAEs in relation to treatment cycle numbers in 109 patients. Abbreviation: ALT, alanine transaminase; AST, aspartate aminotransferase; γ GT, gamma-glutamyltransferase.

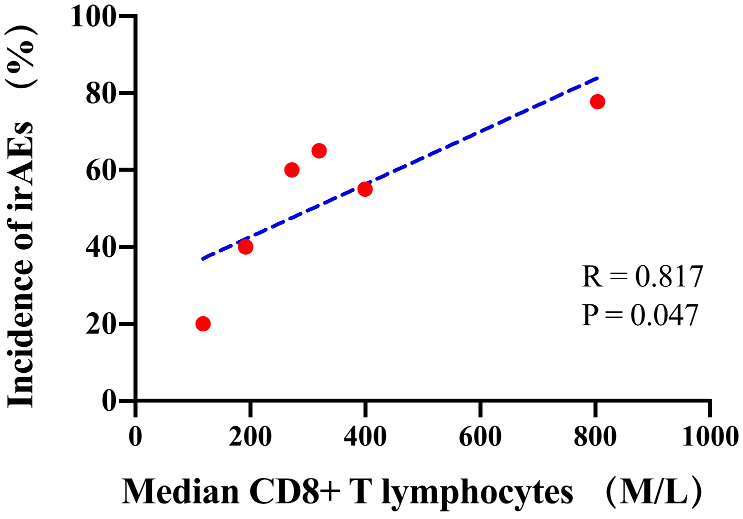

Supplementary figure S3. Association between median baseline level of CD8[+] T lymphocytes and incidence of immune-related adverse events (irAEs) during immunotherapy. The straight line represents the linear fit. Pearson correlation coefficient (R) and the corresponding *p* value are shown at the bottom-right of the figure.
